# Practice Summary of Antimicrobial Therapy for Commonly Encountered Conditions in the Neonatal Intensive Care Unit: A Canadian Perspective

**DOI:** 10.3389/fped.2022.894005

**Published:** 2022-07-08

**Authors:** Joseph Y. Ting, Julie Autmizguine, Michael S. Dunn, Julie Choudhury, Julie Blackburn, Shikha Gupta-Bhatnagar, Katrin Assen, Julie Emberley, Sarah Khan, Jessica Leung, Grace J. Lin, Destiny Lu-Cleary, Frances Morin, Lindsay L. Richter, Isabelle Viel-Thériault, Ashley Roberts, Kyong-soon Lee, Erik D. Skarsgard, Joan Robinson, Prakesh S. Shah

**Affiliations:** ^1^Department of Pediatrics, University of Alberta, Edmonton, AB, Canada; ^2^Department of Pediatrics, University of British Columbia, Vancouver, BC, Canada; ^3^Division of Infectious Diseases, Department of Pediatrics, Université de Montreal, Montreal, QC, Canada; ^4^Department of Pharmacology and Physiology, Université de Montréal, Montreal, QC, Canada; ^5^Division of Neonatology, Department of Pediatrics, University of Toronto, Toronto, ON, Canada; ^6^Department of Pharmacy, Sunnybrook Health Sciences Centre, Toronto, ON, Canada; ^7^Department of Microbiology, Infectious Diseases and Immunology, Université de Montreal, Montreal, QC, Canada; ^8^Division of Neonatology, Department of Pediatrics, McMaster University, Hamilton, ON, Canada; ^9^Division of Neonatology, Department of Pediatrics, University of Manitoba, Winnipeg, MB, Canada; ^10^Department of Microbiology, McMaster University, Hamilton, ON, Canada; ^11^Department of Pediatrics, University of Massachusetts, Worcester, MA, United States; ^12^School of Medicine, Queen's University, Kingston, ON, Canada; ^13^Northern Ontario School of Medicine, Thunder Bay, ON, Canada; ^14^Division of Infectious Diseases, Department of Pediatrics, CHU de Québec-Université Laval, Québec, QC, Canada; ^15^Division of Pediatric Surgery, Department of Surgery, University of British Columbia, Vancouver, BC, Canada; ^16^Division of Infectious Diseases, Department of Pediatrics, University of Alberta, Edmonton, AB, Canada

**Keywords:** neonate, sepsis, urinary tract infection, surgical prophylaxis, ventilator-associated pneumonia, necrotizing enterocolitis, antimicrobial

## Abstract

Neonates are highly susceptible to infections owing to their immature cellular and humoral immune functions, as well the need for invasive devices. There is a wide practice variation in the choice and duration of antimicrobial treatment, even for relatively common conditions in the NICU, attributed to the lack of evidence-based guidelines. Early decisive treatment with broad-spectrum antimicrobials is the preferred clinical choice for treating sick infants with possible bacterial infection. Prolonged antimicrobial exposure among infants without clear indications has been associated with adverse neonatal outcomes and increased drug resistance. Herein, we review and summarize the best practices from the existing literature regarding antimicrobial use in commonly encountered conditions in neonates.

## Introduction

Neonates admitted to the neonatal intensive care unit (NICU) are highly susceptible to overwhelming infections, which can progress rapidly with potentially disastrous consequences. Neonates, especially those born preterm, are highly susceptible to over-whelming generalized infections because of their immature immune system, and the need for invasive devices such as central catheters.

Antimicrobials are commonly prescribed in the NICU (1, 2). However, there is a wide practice variation in the choice and duration of antimicrobial treatment, even for relatively common conditions, due to a lack of evidence-based guidelines (3–8). Here we conducted a narrative review of the best practices regarding antimicrobial use from the existing literature in six commonly encountered conditions in neonatology, namely early-onset sepsis (EOS), late-onset sepsis (LOS), ventilator-associated pneumonia (VAP), NEC, urinary tract infection (UTI), and surgical site infection (SSI) (Table 1).

**Table 1:** Summary of recommendations for agent and duration of antimicrobial therapy for commonly encountered conditions in the NICU.

**Condition**	**Suggested antibiotics and duration**	**Comments**
**Early-onset sepsis**		
Empirical antimicrobial use with negative blood and/or culture	Ampicillin and Gentamicin as empirical choice. To be discontinued in 36–48 h in general	Early cessation of antibiotics to be supported by the clinical and laboratory findings.
Culture-proven bacteremia	7–10 days	
Culture-proven meningitis	14–21 days	14–21 day for meningitis caused by Gram positive organisms; at least 21-day recommended for *E. Coli* and other meningitis caused by Gram-negative bacilli
**Late-onset sepsis**		
Empirical antimicrobial use with negative blood and/or culture	Cloxacillin & Gentamicin or per local antibiogram/ patient characteristics. To be discontinued in 36–48 h	Early cessation of antibiotics to be supported by the clinical and laboratory findings.
Culture-proven bacteremia	7–14 days	14-day for *S. aureus* bacteremia; timely removal of catheter being the key to reduce treatment failure
Culture-proven meningitis	14–21 days	14–21 day for meningitis caused by Gram positive organisms; at least 21-day recommended for *E. Coli* and other meningitis caused by Gram-negative bacilli
**Ventilator-associated pneumonia (VAP)**		
VAP	7–8 days	Longer treatment duration for those with complicated VAP or secondary bacteremia.
**Necrotizing enterocolitis (NEC)**		
NEC	Ampicillin and gentamicin ± metronidazole or clindamycin; Piperacillin-tazobactam as a single agent Stage I: 3 days Stage II: 5–7 days Stage III 10–14 days	In case of intra-abdominal abscesses, antibiotics should be continued until clinical and radiological responses are established.
**Urinary tract infection (UTI)**		
UTI	5–7 days of parenteral therapy	Oral therapy is not recommended for premature neonates.

## Importance of Antimicrobial Stewardship in the Neonatal Populations

Antimicrobials are commonly prescribed for treatment or prophylaxis purposes in the NICU, since the clinical signs and symptoms of infection are often non-specific and difficult to differentiate from common non-infectious pathologic processes (1). Furthermore, early decisive treatment with broad-spectrum antimicrobials is the preferred clinical choice for treating sick infants with possible bacterial infection. Since it is often impossible or even ethical to conduct blinded randomized controlled trials on the choice or duration of antibiotics for critically ill infants, recommendations are often derived from combining center-specific data on the most common infecting organisms to make the most rationale choices for empiric therapy. Therapy duration has been shown to not correlate with clinical findings or risk index (9–12). Variability in antimicrobial prescribing across sites has been reported even after adjustment for patient characteristics correlated with illness severity (13).

Exposure to broad-spectrum antibiotics can lead to gut dysbiosis, adverse neonatal outcomes, (14–19), and emergence of multi-drug resistant organisms (MDRO) such as bacteria producing extended-spectrum β-lactamases (ESBL) (20, 21), vanco-mycin–resistant *Enterococcus*, (22) carbapenem-resistant *Enterobacteriaceae*, (23, 24) as well as invasive Candidal infection (17, 25). Prolonged antimicrobial exposure without culture-proven sepsis or necrotizing enterocolitis (NEC) has been associated with increased mortality, morbidities, and possibly worse early neurodevelopmental outcomes in preterm infants (18, 26–28).

Antimicrobial stewardship programs (ASP) optimize clinical outcomes while mitigating unintended consequences of antimicrobial misuse for control of emergence of MDRO (29). ASP includes active monitoring for antimicrobial resistance, fostering of appropriate antimicrobial use, and collaboration with an effective infection control program and pharmacy department to minimize secondary spread of resistance (30). There is currently lack of NICU-specific antimicrobial stewardship best practices and strategies that address the unique challenges faced in the management of NICU patients (31, 32). Moreover, there are no established sets of evidence-based NICU-specific metrics to measure the success or failure of ASP initiatives (31).

Review of the current best evidences in commonly encountered neonatal conditions sets the stage for the establishment of a proper NICU-specific ASP to promote the judicious use of antimicrobials.

## Early Onset Sepsis

EOS is a significant contributor to neonatal mortality and other adverse outcomes (33). Antibiotics are administered to approximately 2–15% of term newborns and 75–95% of preterm newborns owing to the risk of EOS (34). In the Canadian Neonatal Network, EOS refers to culture-proven sepsis or meningitis within the first two calendar days of life (35). The Center for Disease Control and Prevention (CDC) defines EOS as blood and/or cerebrospinal fluid (CSF) culture-proven infection in newborns occurring at less than 7 days of life (36). Preterm EOS is defined as the isolation of a pathogenic bacterial species from a blood or cerebrospinal fluid culture obtained within 72 h after birth (37, 38).

### Epidemiology and Microbiology

EOS mainly develops from the transmission of bacteria that colonize the maternal genito-urinary and gastrointestinal system during the intrapartum period (39). Group B *streptococcus* (GBS) and *Escherichia coli* account for 70–80% of blood and cerebrospinal fluid cultures (40–42). Intra-partum antimicrobial prophylaxis (IAP) became the standard of clinical care to prevent GBS transmission in the 1990s (43). With its wide implementation, the incidence of EOS due to GBS in the United States has decreased from 1.5–1.7 cases per 1000 live births to 0.23 per 1,000 live births (44). Recent population surveillance studies in North America revealed that *E. coli* disease primarily occurs among preterm infants; GBS disease primarily occurs among term infants, with almost half occurring in infants born to mothers with negative GBS screening test results (45).

### Predictors of Development of EOS

Gestational age (GA) is the strongest predictor of EOS (39). The incidence of EOS is inversely associated with GA, with 0.5 cases per 1000 neonates born at ≥34 weeks' gestation and up to 32 cases per 1,000 in those born at 22–24 weeks of gestation (39, 46). The EOS rate was reported to be 4.3–5.2% among infants born at a GA of <25 weeks in Canadian cohorts (47, 48). The risk factors associated with EOS in term infants include intrapartum GBS colonization or GBS bacteriuria during the current pregnancy, previous infant with invasive GBS disease, prolonged rupture of membranes ≥18 h, and maternal temperature ≥38°C (49, 50). In preterm infants (≤35 weeks' gestation), unexplained spontaneous preterm labor, premature rupture of membranes (PROM), acute onset of atypical fetal heart rate, or concern for intra-uterine infection (or chorioamnionitis) are risk factors for EOS (39).

### Diagnostic Tools

Infants often have nonspecific clinical manifestations that can be indistinguishable from normal newborn transitions or prematurity conditions (38). Positive blood or cerebrospinal fluid culture is the hallmark of EOS. In general, blood cultures growing clinically significant pathogens are positive within 24–36 h of incubation (51, 52). Molecular assays like real-time PCR have been evaluated for the rapid detection of pathogens in neonatal EOS. They provide care providers with the critical information of organism identification and antimicrobial susceptibility from cultures flagged as positive, faster than with traditional microbiology methods, which may allow the clinicians to narrow empiric antibiotic choices more efficiently. They are also being studied as 'add-on' tests to identify pathogens that are not detected by conventional blood or CSF culture (53, 54).

Low white blood cell counts, low absolute neutrophil counts, and high immature-to-total neutrophil ratios are associated with increased odds of infection, but the sensitivities of complete blood count (CBC) indices were low (0.3–54.5%) to rule out EOS (55, 56). There is concern regarding falsely normal white blood cell screening tests among infants with fulminant sepsis, particularly when the CBC screening is performed between 1 and 7 h of life; consequently, repeated CBC screenings between 12 and 24 h of age are reasonable (57, 58).

C-reactive protein (CRP) is a non-specific inflammatory marker that typically begins to increase 6–8 h after infection onset. Levels are only elevated in 35–55% of neonates at the onset of illness, and for the first few hours after birth (59, 60). It is sensitive but not specific, so if it remains normal at 24–36 hours and the infant appears well, antibiotics can be safely discontinued (61, 62).

In the Neonatal Procalcitonin Intervention Study (NeoPInS), Procalcitonin-guided decision making was found to be superior to standard care in reducing antibiotic therapy in neonates with suspected EOS (intention to treat: 55.1 vs. 65.0 h, *p* < 0.0001) (63). Dongen et al. reported that umbilical cord blood procalcitonin (PCT) levels are increased in newborns ≥32 weeks with proven or probable EOS, but PCT might not be a reliable marker after maternal antibiotic treatment (64).

Cytokines, including interleukin 6 (IL-6), interleukin 8 (IL-8), gamma interferon (IFN-γ), and tumor necrosis factor alpha (TNF-α), and cell surface antigens such as soluble intercellular adhesion molecule (sICAM) and CD64, have been studied as measures for neonatal sepsis; however, the heterogeneity of available evidence prevents conclusions regarding their routine use as definitive diagnostic tools (38, 65, 66).

There is no additional benefit to include urine microscopy or culture as part of the investigations for early-onset neonatal infection in the first 24 h of life among both preterm and term infants because most infections of the urinary tract in this population are secondary to hematogenous seeding of the kidney by bacteremia (67, 68).

While lumbar puncture (LP) is an important means of obtaining CSF to rule out the presence of meningitis in infants, its routine use in neonates for EOS evaluation remains controversial (38). In any infants, the timely collection of CSF samples should balance the cardiorespiratory stability of the infant, the risk of EOS, and the potential harm associated with delayed antibiotic therapy. While antibiotics can render the CSF culture negative and no single CSF parameter can reliably exclude the presence of meningitis, antibiotic initiation should not be delayed by the LP procedure itself in critically ill infants (69).

### Risk Assessment for EOS in Infants Born at ≥35 Weeks' Gestation

The primary focus is to identify infants at high risk for infection to warrant empiric antibiotic therapy. Three approaches currently exist in the literature for the use of risk factors to identify infants who are at increased risk of EOS (50).

In the first approach, the following threshold values for intrapartum risk factors are utilized: (i) any newborn infant who is ill appearing; (ii) a mother with a clinical diagnosis of chorioamnionitis; (iii) a mother with GBS colonization and who received inadequate IAP, with a duration of ROM of >18 hours or birth before 37 weeks of gestation; or (iv) a mother with GBS colonization and who received inadequate IAP but with no additional risk factors. Recommendations include observation in the hospital, laboratory testing, empirical antibiotic therapy for infants, or a combination of these (50, 70).

In the second approach of the multivariate risk prediction model, an individualized risk of EOS based on several risk factors is generated, and then that risk is modified depending on the infant's clinical condition specified by explicit clinical characteristics in the first 12 h after birth (34). The Kaiser Permanente Research group developed an online sepsis risk calculator (SRC) to evaluate the risk of neonatal EOS, which has been validated as a safe way to reduce the proportion of infants receiving empiric antibiotics without apparent adverse effects in infants born after ≥35 weeks' gestation (71).

The final approach consists of serial evaluations within the first 48 hours of age for clinical signs of illness among infants with risk factors to identify infants with EOS, which have been shown to significantly decrease with the use of laboratory tests, blood cultures, and empirical antibiotic agents (50, 72).

Canadian Pediatric Society has also developed an algorithm to provide updated recommendations for the care of term (≥37 weeks' gestation) newborns with risk factors for EOS during the first 24 h of life (73).

The above mentioned approaches and algorithms must be used in conjunction with meticulous history-taking, careful clinical examination, and serial assessment of the at-risk infant. Used in this way, clinicians can identify those infants who are at high risk of developing EOS and prevent unnecessary antibiotic exposure in a substantially larger number of infants who are uninfected.

Blood culture is the gold standard for diagnosis of EOS, but its sensitivity can be affected due to small volume of blood sample and antibiotics given to newborn before sampling. In the recent years, umbilical cord blood culture for the diagnosis of EOS in term infants has been advocated as a large volume can be readily obtained and shown to have >80% sensitivity and specificity compared to venous blood samples (74), though strict and proper sterile techniques are needed to prevent contamination (75).

### Risk Assessment for EOS in Infants Born at ≤34 Weeks' Gestation

GA is the strongest predictor of EOS, and two-thirds of preterm births are associated with preterm labor, PROM, or clinical concern for intrauterine infection (49). Clinical features and common laboratory tests, such as CBC and CRP, are impacted by maternal conditions and cannot be reliably used to predict EOS among preterm infants. The objective of EOS risk assessment among preterm infants is to determine which infants are at the lowest risk for infection such that they are eligible to be spared from the administration of empirical antibiotics, or at the very least to be administered a shorter duration of empirical antimicrobial coverage (39).

Preterm infants who are considered at a lower risk for EOS have the following characteristics at the time of delivery: (i) obstetric indications for preterm birth (e.g., maternal preeclampsia or other noninfectious medical illness or placental insufficiency); (ii) cesarean birth; and (iii) absence of labor, attempts to induce labor, or any ROM before delivery (76, 77). Infants fulfilling these criteria can be considered at the lowest risk of EOS and may be managed without empiric antibiotics at birth (39). In a single-center study evaluating the impact of such a risk-factor approach in EOS evaluation among preterm infants, the guideline implementation was associated with decreased antibiotic initiation among low-risk, extremely low birth weight (ELBW) infants, without any change in incidence of confirmed infection or death (78). A randomized controlled trial is currently being conducted to test if the incidence of adverse outcomes is higher in babies receiving empiric antibiotics in the first week of life compared to babies receiving placebo (79). This study targets a population of ELBW infants that are clinically stable that did not have a known exposure to intraamniotic infection and were not born preterm for maternal indications.

In contrast, infants born preterm because of maternal cervical incompetence, preterm labor, PROM, clinical concern for intra-amniotic infection, or acute onset of unexplained non-reassuring fetal status are at the highest risk for EOS. All should undergo EOS evaluation with blood culture and empirical antibiotic treatment (39). Clinicians often express concerns about poor sensitivity of blood cultures in neonates resulting from inadequate inoculant volumes, resulting in frequent diagnosis of “culture-negative sepsis”, and prolonged use of empiric antibiotics in neonatal units (80). To help avoid a “culture-negative sepsis” diagnosis and prolonged use of unnecessary empiric antibiotics in neonates, a minimum of 1 ml of blood culture volume has been recommended, based on the bench study and expert recommendations (80–83). Real-time PCR has the advantage of detecting organisms present in low concentrations using small volumes of blood and providing rapid results. However, the sensitivity and specificity for diagnosis of EOS and detection of antibiotic resistance remains under investigation (53, 84).

### Empirical Antimicrobial Therapy of EOS

The combination of ampicillin and gentamicin is most commonly administered to neonates at risk for EOS. This combination targets the most common pathogens pending culture results (39, 50). Aminoglycoside-based regimens should generally be used instead of cefotaxime-based treatments because of lower levels of susceptibility to cefotaxime and to potentially prevent the development of antibiotic resistance (85).

The empirical administration of more broad-spectrum agents may be indicated in infants who are critically ill with additional risk factors, particularly after prolonged antepartum maternal antibiotic treatment, or if the mother is known to be colonized with an antimicrobial resistant organism, until appropriate culture results are known (39, 50). In some places, benzylpenicillin is being used instead of ampicillin, which is a narrower-spectrum alternative. Decisions regarding the optimal empirical coverage should be guided by the local epidemiology and antibiogram.

### Importance of Judicious Antimicrobial Use in Early Life

The use of the narrowest-spectrum effective antimicrobials allows for the targeted killing of the infecting pathogen while minimizing undesirable impacts on the colonizing microbial flora of the infants. At the individual level, antibiotic use either perinatally or postnatally has been linked to disruptions in the microbiome (86, 87). Alteration in intestinal microbiota has been postulated to play a critical role in the development of significant neonatal morbidities, through the regulation of systemic inflammation (19). Antibiotic treatment in the first week after birth may also increase the subsequent risk of wheezing, infantile colic, and childhood obesity (88, 89). A study from the Canadian Neonatal Network demonstrated that a prolonged initial antibiotic exposure of >3 days within the first week after birth was associated with increased odds of mortality or significant morbidities, including chronic lung disease, patent ductus arteriosus, retinopathy of prematurity, and severe neurologic injury, even after adjustment for baseline differences in the characteristics of very-low-birth-weight (VLBW) infants (26).

Empirical cefotaxime use without justification should be avoided, as its overuse may increase the prevalence of MDRO, candidiasis, and possibly neonatal death (20, 21, 90, 91).

### Re-evaluation for the Continuation of Antibiotic Treatment 36 h After Initiation

In the absence of positive blood culture or clear evidence of site-specific infection, infants that were administered empirical antibiotics because of risk factors for EOS or clinical indicators of possible infection who remain well should have their antibiotics discontinued after 36 h, particularly when the trends of CRP concentration are reassuring (39, 50, 65, 92). If antibiotics are continued for longer than 36 h despite negative blood cultures, the infant should be reexamined at least once every 24 h to determine whether it is appropriate to stop the antibiotic treatment (92).

Persistent cardiorespiratory instability is common among preterm infants with VLBW and this symptom is not by itself an indication for prolonged empirical antibiotic administration (39). Continuing empirical antibiotic administration in response to laboratory test abnormalities is rarely justified, particularly among preterm infants at a lower risk for EOS and those born with maternal obstetric conditions known to affect fetal hematopoiesis (39).

There have been concerns regarding the incomplete detection of low-level bacteremia and the effects of intrapartum antibiotic administration; modern blood culture systems can reliably detect low levels of bacteremia provided that 1 mL of blood is inoculated, and studies have shown no impact of IAP on time to detection of EOS (51, 93, 94).

### Duration of Therapy for Positive Blood Culture

When EOS is confirmed by blood culture, a lumbar puncture should be performed if not done previously (39). Antibiotic choice should be pathogen-specific with a narrow effective spectrum of activity and should be guided by sensitivity results. There are a few studies that have guided the duration of therapy. The duration of antimicrobial therapy for uncomplicated bacteremia without a focus is usually 7–10 days with a pathogen-specific antibiotic (95). Seven days may be considered for some low-risk cases of neonates over 32 weeks of gestation and weighing over 1500 g (96–98). When uncomplicated meningitis attributable to GBS is diagnosed, therapy is extended to 14 days (95). Extended courses of treatment are indicated for complications, including cerebritis and osteomyelitis. Antimicrobial therapy is usually recommended to be administered for a minimum of 3 weeks for gram-negative meningitis, provided sterility of the CSF is ensured 24-48 hours after the appropriate treatment commences (99–101).

### Prevention

IAP for at-risk women has become the standard of care in many countries over the past two decades and is an effective way to prevent EOS (102–104). A meta-analysis revealed that compared with a risk-based strategy, screening-based prophylaxis was associated with a reduced risk of GBS-EOS (105). The Society of Obstetricians and Gynecologists of Canada (SOGC), in 2018, reaffirmed the recommendation of offering all women screening for colonization with GBS at 35–37 weeks of gestation, by performing culture tests with one swab from the vagina and one from the rectum (106).

### Practice Summary

- GBS and *E. coli* are the most common organisms responsible for EOS in term and preterm infants, respectively.
- CBC-derived index or CRP cannot reliably rule out EOS.
- Ampicillin and gentamicin in combination have good efficacy for most causative organisms in EOS, although the choice of therapy should be guided by local antibiotic susceptibility data.
- Unless there is clear evidence of sepsis or site-specific infection, empirical antibiotic therapy should be discontinued after 36 h if the blood culture is negative, particularly when there are no other markers of infection (e.g. C-reactive protein).
- Preterm infants at a lower risk for EOS may be managed without empiric antibiotics at birth.
- The judicious use of antibiotics should be promoted to decrease the risks associated with prolonged antibiotic administration in the face of sterile cultures and with the use of unnecessary broad-spectrum therapy.

## Late Onset Sepsis

In general, LOS refers to an invasive infection occurring in neonates older than 3 days of life, indicating postnatal (hospital or community) acquisition (107–109). It can lead to life-threatening organ dysfunction caused by a dysregulated host response to infection (110, 111). LOS accounts for a significant proportion of morbidity and mortality in neonates and may adversely affect brain development and lead to neurodevelopmental impairment among survivors (33, 112).

### Epidemiology, Microbiology, and Risk Factors

The two major risk factors that predispose neonates in the NICU to sepsis are preterm birth and low birth weight (111). In the Canadian Neonatal Network, a 5% incidence of LOS has been reported among neonates with a BW of 1000–1499 g, who survived for more than two calendar days, but the incidence increased to 30–36% in those with a BW of 750–999 g (47, 48). Other known risk factors for LOS include prolonged mechanical ventilation, use of intravascular devices, failure of early breast milk feeding, and prolonged hospitalization (111, 113, 114). Central line–associated bloodstream infections (CLABSIs) are the most common type of LOS among infants in the NICU, resulting from extraluminal or intraluminal catheter contamination after colonization of the neonatal skin, oropharynx, intestinal tract, and/or the catheter surface (115).

LOS can result from viral, bacterial, or fungal microorganisms (111). In infants admitted to NICUs, coagulase-negative *Staphylococcus* (CoNS) is the most common pathogen in LOS (116). Other common bacteria include *Staphylococcus aureus, E. coli, Klebsiella spp, Enterococcus spp*, and GBS (116). Neonates are also susceptible to invasive infections from organisms found in the hospital environment, such as *Enterobacter spp, Pseudomonas spp*, and *Serratia spp*. Fungi, specifically yeasts as *Candida spp*., can also cause systemic infections; however, its incidences varied across different NICUs (117).

### Diagnostics

The gold standard for the diagnosis of LOS is a positive blood culture. However, limitations to positive blood culture include the delay between collection and positivity, false positive results from contamination, and false negatives resulting from antibiotic therapy prior to collection or submission of inadequate blood volume (116). A minimum of 1 mL of blood should preferably be obtained from two different venipuncture sites (83, 118). This minimum volume is recommended due to the potential for paucibacillary infection (1–10 colony-forming units (CFU) per milliliter), with a reported sensitivity of 98% for volumes >1 mL (119). In adults, if an intravascular catheter is in place, peripheral and central blood cultures can improve the diagnosis of central line-associated bloodstream infection based on differences in time to positivity of culture from the two sites of >2 h and may also aid in the identification of sample contamination (120). This concept has not been studied in the neonatal population, likely because of the relatively small-sized lumen of their central venous catheters.

Neonatal meningitis frequently occurs in the absence of bacteremia in patients with LOS (69). Conducting LP is important in the diagnosis of meningitis, although its interpretation can be affected by the high frequency of traumatic LPs and the variability of cytologic and biochemical indices in the neonatal population (121). Repeated CSF fluid parameters may provide additional insights into the treatment response and adverse effects from the outcomes of bacterial meningitis (122). Bacterial nucleic acid detection in CSF (such as 16s ribosomal RNA) can be useful if CSF culture results are negative because of preceding antibiotic therapy. Urine examination is also an important component of LOS evaluation, as UTI is not uncommon, particularly among preterm infants [Please refer to Section 6 on “urinary tract infection” for diagnostics, interpretation, and management].

Due to non-specific symptomatology, biomarkers are used in addition to microbiologic cultures to guide the initiation and duration of antibiotic therapy (123, 124). There are numerous sepsis biomarkers like chemokines, cytokines, leucocyte cell surface antigens, and acute phase proteins described, however, none of these are considered standard of care at the bedside (125). Procalcitonin and CRP are the most extensively studied biomarkers and appear to have the greatest diagnostic utility at 12–24 h (mid-phase) and >24 h (late phase) from initial clinical suspicion of LOS (123, 124). Single values of CRP or procalcitonin are neither sensitive nor specific to guide care decisions in the diagnosis of sepsis. Two CRP levels (<10 mg/L obtained at 24 hours apart and at 8–48 h after onset of possible LOS) indicated that bacterial infection is unlikely (60).

### Empirical Antimicrobial Therapy of LOS

Empiric antimicrobial therapy should be guided by local epidemiology (e.g., methicillin-resistant *Staphylococcus aureus* (MRSA) incidence) and antibiogram data, including coverage for common gram-negative and gram-positive pathogens (111). One must also consider the pharmacokinetic and pharmacodynamic properties of antibiotics in neonates (126). Cloxacillin, a narrow-spectrum antibiotic, is recommended to treat gram-positive LOS, especially if *S. aureus* infection is suspected. Vancomycin—a broad-spectrum antibiotic—is now discouraged as empiric LOS therapy, if the prevalence of MRSA in the community remains low, as it then becomes difficult to determine whether blood cultures positive for CoNS are contaminated (127). No survival benefit with empirical vancomycin therapy has been demonstrated for CoNS bloodstream infections in infants from a retrospective review including 4,364 infants from 348 NICUs, even though the median duration of bacteremia was 1 d longer in infants who received delayed vancomycin therapy (128).

Unless meningitis is suspected, gram-negative coverage with an aminoglycoside is preferred over third-generation cephalosporins because of the potential for the latter to induce antimicrobial resistance. In most jurisdictions, aminoglycosides are more likely to cover nosocomial gram-negative bacteremia (e.g., *Enterobacter spp, Serratia spp, Pseudomonas spp*) (129).

### Duration of Antimicrobials for Established Bacteremia and Bacterial Meningitis

Currently, there are no evidence-based guidelines on the duration of antimicrobial treatment for neonatal bacteremia and meningitis. The duration is determined by each patient's risk factors, clinical evaluation, laboratory tests, specific pathogens that are identified, and their susceptibility to antimicrobials (111, 130).

For bacteremia, in general, the recommended duration of antibiotics ranges from 7 to 14 days, depending on the pathogen isolated. Using CRP and blood cultures as a measure, it was found that a 10-day antibiotic duration was as effective as 14 days in neonatal bacteremia (97). In another study, 69 neonates with septicemia were randomized to receive a 7- or 14-day course of antibiotics (98). Although the 7-day antibiotic treatment appeared to be sufficient for other pathogens, neonates with S. *aureus* bacteremia experienced more treatment failure with 7 days of treatment compared to that with 14 days of treatment, which thus remains the recommended duration (98). Of note, retention of the central venous catheter in neonates with bloodstream infection is associated with delayed resolution of bacteremia and a higher incidence of recurrence (131).

The treatment duration for meningitis has traditionally ranged from 14 to 21 days, depending on the pathogen isolated. For example, 14 days of treatment is recommended for uncomplicated GBS meningitis, whereas a minimum of 21 days is recommended for uncomplicated meningitis caused by gram-negative bacteria (132, 133). However, it is important to note that the treatment duration should ultimately be individualized to the pathogen and the patient's clinical response.

### Management of Culture-Negative Sepsis

Culture-negative sepsis implies that a neonate is clinically suspected to have sepsis, often due to nonspecific symptoms; therefore, a complete course of antibiotics is prescribed despite sterile cultures. As aforementioned, sterile cultures may be falsely negative owing to low-level bacteremia, antibiotic pre-treatment, or bacterial infection at a site that is not readily cultured (such as the lung) (134). As per recent reports, up to ten-fold higher antibiotic utilization for culture-negative sepsis is observed than that for culture-proven sepsis, (127, 135) however, this varies widely between institutions. Empirical therapy should be limited to 24–36 h if the infant shows no subsequent clinical or laboratory evidence of infection and cultures are negative (126).

Prolonged antibiotic courses (>5 days) in culture-negative settings have been associated with increased odds of necrotizing enterocolitis (NEC) and death (4% increased risk with each additional day) (136). Unnecessary antimicrobial use is increasingly recognized to pose significant harm to NICU patients, including increased risk of invasive candidiasis, prolonged hospitalization, bacterial resistance, (137) and predisposition to inflammatory dysregulation with long-term adverse health outcomes (138).

### Practice Summary

- Blood cultures are the gold standard for diagnosis of LOS. Every effort should be taken to optimize the sensitivity and specificity of blood culture (1 mL is considered the minimal volume).
- Normal values of serial CRP obtained at 24 h apart and at 8–48 h after the onset of possible LOS indicated that bacterial infection is unlikely.
- For LOS without concerns of meningitis, cloxacillin, and gentamicin are the recommended antibiotics to provide empirical coverage for *S. aureus* and most gram-negative bacteria, considering the local epidemiology and antimicrobial resistance risk factors.
- For patients with clusters of gram-positive cocci or CoNS as the preliminary finding in blood cultures, a second culture should be obtained prior to initiating vancomycin to reduce the overtreatment of potential CoNS-contaminated cultures.
- The duration of therapy is dependent on the type of pathogens and infectious complications, but generally 7 to 14 days are adequate for neonatal bacteremia.
- Retention of the central venous catheter in neonates with bloodstream infection can be associated with delayed resolution of bacteremia and a higher incidence of recurrence.
- Prolonged antibiotic courses (especially >5 days) in culture-negative settings have been associated with adverse neonatal outcomes and are thus discouraged.

## Ventilator-Associated Pneumonia

Neonatal ventilator-associated pneumonia (VAP) is an important healthcare-associated infection (HAI) with significant morbidity, ranging from 1.1 to 10.9 episodes per 1,000 ventilator-days, and remains a leading cause of antibiotic use in the NICU (139–142). Quality improvement efforts are challenging from many perspectives, including surveillance, diagnostic accuracy, and optimal therapy. Barriers to improvement in neonatal VAP practices include the lack of standardization or guidelines for definition and surveillance, over-reliance on microbiological results, uncertainty regarding the choice of empiric antibiotics, and duration of antimicrobial treatment (143–147). These barriers result in major practice variations and antimicrobial overuse in this era of heightened antimicrobial stewardship efforts.

### Definition and Surveillance

Historically, the CDC definition of VAP for infants up to one year of age was the most widely used definition for neonates, but this definition was criticized for its diagnostic inconsistency, especially for preterm neonates (148). Initiatives to refine the CDC definition of VAP have led to the introduction of the term pediatric ventilator-associated condition (VAC) by the National Health and Safety Network (NHSN). Pediatric VAC has an objective definition for respiratory deterioration defined as increase in mean airway pressure ≥4 mmHg and/or FiO_2_ ≥0.25 for a specified duration of ≥2 calendar days (149). As pediatric VAC is not specific for infectious ventilator events, additional clinical and management criteria are required to fulfill the conditions for infection-related ventilator-associated complication (IVAC), and additional microbiological criteria are required to fulfill the criteria for possible VAP (150). Chest radiograph findings have been removed from the NHSN criteria for VAC, IVAC, and possible VAP for all populations due to concerns regarding reliability, which is particularly relevant in that the interpretation of radiological changes is especially challenging in neonates with pre-existing lung pathologies (148, 151). Quality improvement initiatives that target VAP, using internally consistent definitions, have been successful in improving practices (148–157) and are, therefore, encouraged (148).

### Microbiology

Purulence defined as ≥25 polymorphonuclear leukocytes per low power field on tracheal aspirates (TA) and bronchioalveolar lavage (BAL) microscopy has been correlated with the duration of mechanical ventilation and the incidence of bronchopulmonary dysplasia, but not with clinical symptoms (146, 147, 158–161). Similarly, time since intubation is the strongest predictor of positive TA quantitative cultures, with approximately half of the bacterial growth occurring after 4–10 days of mechanical ventilation and almost 100% after 10 days (148, 161, 162). Most positive TA cultures represent endotracheal tube colonization and oropharyngeal contamination rather than infection (147, 148, 160) as supported by the fact that up to 58% of TA cultures are polymicrobial (139). Thus, a positive microbiological result is only one of several criteria for VAP. Certain groups have reported that less invasive BAL (“mini-BAL”) is safe in preterm infants and may decrease the false positive rates in VAP diagnosis (140, 158, 160).

### Antimicrobial Treatment – Choice and Duration

The choice of empirical therapy should be made after considering the severity of illness, prior colonization, local antibiogram data, and risk factors for multidrug-resistant (MDR) pathogens (163). The most common pathogens for VAP are *S. aureus* and Enterobacteriaceae, and these organisms should be the target of empirical antimicrobials, whereas *Pseudomonas aeruginosa* should be targeted only in NICUs with a high prevalence of this pathogen. Narrow-spectrum therapy (e.g., cefazolin or a combination of cloxacillin plus either an aminoglycoside or cefotaxime) should be favored in non-critically ill neonates with no prior history of colonization with resistant bacteria, and in NICUs with low rates (<10%) of multidrug-resistant pathogens (164, 165).

After initiation of empirical therapy for possible VAP, antimicrobial agents should be reassessed 24–36 h after their initiation (148, 166). If an alternative diagnosis is established that explains the patient's clinical or radiological deterioration, or if TA cultures are negative, antimicrobial therapy for VAP should be discontinued. In neonates with probable VAP, the antimicrobial spectrum should be narrowed based on the culture results (164, 167). Combinations of antibiotics are not recommended as this does not hasten clinical recovery.

The optimal treatment duration for VAP in neonates is unknown (168). However, some key principles, such as time to clinical improvement, microbial burden/source control, and host immune status should be considered (169). A meta-analysis of four randomized controlled trials in immunocompetent adults suggests that a 7-to 8-day antibiotic treatment, irrespective of the causative pathogen, is as effective as a 15-day course in terms of clinical cure, mortality, and relapse risks, and is associated with a lower risk of subsequent MDR infections (164, 170). Treatment duration should be longer for those with complicated VAP (e.g., pneumatocele, lung abscess, or empyema), VAP with secondary bacteremia, and for those with underlying comorbidities impacting immunological response.

## Practice Summary

- Definitions for neonatal VAP should be internally consistent and align with external standards to allow for future benchmarking. A dedicated multidisciplinary team that includes neonatology and infectious disease specialists with VAP expertise should review all possible VAP cases. Due to high rates of colonization/contamination, positive microbiologic results should not be the major consideration for defining VAP. An objective review of all clinical criteria is required to determine the pathogenicity of bacteria grown from tracheal secretions.
- Narrow-spectrum therapy should be favored in non-critically ill neonates with no prior history of colonization with resistant bacteria.
- After initiation of empirical therapy for possible VAP, antimicrobial agents should be reassessed 24–36 h after their initiation. If an alternative diagnosis is identified to explain the patient's clinical or radiological deterioration, or if tracheal aspirate cultures are negative, antimicrobial therapy that is specifically targeting VAP should be discontinued.
- The optimal treatment duration for VAP in neonates is unknown, although a 7- to 8-day antibiotic treatment may be reasonable based on the limited existing literature.

## Necrotizing Enterocolitis

Necrotizing enterocolitis (NEC) is a leading cause of neonatal morbidity, affecting approximately 1 in 1000 live births (171). Premature infants are especially at risk, with an incidence of up to 2.5 and 4.5% in low birth weight (BW) and VLBW infants, respectively (172). Classical management plans include bowel rest, supportive care, antibiotics, and surgery, when necessary. Despite optimal care, outcomes can be poor with high mortality rates and serious complications such as intestinal strictures, short bowel syndrome, or neurodevelopmental impairment (172–179).

### Definition

Multiple risk factors are involved in the pathogenesis of NEC, including intestinal inflammation, ischemia, and bacterial overgrowth in a highly vulnerable gastrointestinal tract (180). Bacterial overgrowth may progress to bacterial translocation across the intestinal wall and intra-abdominal infection with localized or diffused peritonitis, which can lead to bowel perforation (181, 182). Spontaneous intestinal perforation (SIP) is different from NEC. SIP generally occurs in the first week of age in very preterm infants and is not discussed in this narrative review (183).

Diagnosis and staging rely on a combination of clinical, laboratory, and radiologic findings, typically using the modified Bell's staging criteria (184). Bell stage I or suspected NEC presentation is non-specific, and symptoms include feeding intolerance, abdominal distension, and occult blood in the stool. Stage II NEC presents with ileus, dilated bowel loops, grossly bloody stools, and *pneumatosis*. Severe NEC or stage III is characterized by respiratory and metabolic acidosis, a fixed bowel loop, and may progress to intestinal perforation (184, 185).

### Microbiology

Numerous pathogens are associated with NEC. For most infants, blood and peritoneal fluid cultures remain negative, but intra-abdominal polymicrobial infections are assumed to be present (186–189). Since NEC is associated with local bacterial overgrowth of endogenous intestinal flora, antibiotic empirical therapy should target enteric gram-negative aerobic and facultative anaerobic bacilli such as Enterobacteriaceae (e.g., *E. coli* and *Klebsiella* species), enteric gram-positive streptococci (e.g., *Streptococcus anginosus)*, and in some situations, obligate anaerobic bacilli (e.g., *Clostridium perfringens* and *Bacteroides fragilis*) (190–192).

### Antimicrobial Treatment – Choice and Duration

There is a wide variability in empiric antibiotic regimens used in clinical practice to treat NEC, reflecting the lack of clear guidelines on the type and duration of antibiotic treatment (175, 189, 193). The Surgical Infection Society and the Infectious Diseases Society of America (IDSA) published the only guideline addressing this issue in 2010 (190); it mainly discusses the treatment of complicated intra-abdominal infections (cIAI) in adults and older children. For NEC specifically, IDSA recommendations are based on experts' opinions and include the following combinations of broad-spectrum antibiotics: (i) ampicillin, gentamicin, and metronidazole, (ii) ampicillin, cefotaxime, and metronidazole, or (iii) meropenem (190).

To date, published studies have failed to demonstrate the optimal antimicrobial regimen for NEC in terms of efficacy and safety (190, 194). A Cochrane systematic review included two randomized controlled trials (RCT) conducted in the 1980s (194). The first RCT compared ampicillin-gentamicin to ampicillin-gentamicin-clindamycin in 42 premature infants with NEC (195); it showed no beneficial effect on mortality or intestinal perforation with the addition of clindamycin, but found a significantly longer time to successful reinstitution of enteral feeding and a higher incidence of stricture formation in the clindamycin group (195). The other RCT included 20 infants and showed no difference in outcomes with enteral vs. parenteral gentamicin when administered in combination with parenteral ampicillin and gentamicin (196).

More recently, a large multicenter, partly randomized controlled trial was completed with 128 infants selected randomly, and an additional 52 infants were enrolled on a non-randomized basis (197). All infants <121 days of age (≤33 weeks GA) had a cIAI and received one of the following three antibiotic combinations: ampicillin/gentamicin/metronidazole; ampicillin/ gentamicin/ clindamycin; or piperacillin-tazobactam/ gentamicin within 48 h of diagnosis. Mortality assessed at 30 days was similar in all three arms at 8–9%. Adverse events up to 90 days follow-up, events of special interest (including gastrointestinal surgeries, progression to a higher stage of NEC, intestinal stricture or perforation, feeding intolerance), and therapeutic success on day 30 were also similar among the three groups. This large RCT does not support the choice of one regimen over another for the treatment of infants with cIAI.

Finally, several observational retrospective studies have compared antibiotic regimens for NEC management. Most were single-center studies with a limited sample size (189, 193, 198, 199). The largest of all observational retrospective studies involved 2,780 infants with <1500 g BW and diagnosed with NEC from 348 centers (175). After propensity score matching, the results suggest that for infants with NEC that were managed without surgery (stage II), anaerobic coverage was not associated with lower mortality, but resulted in an increased risk of intestinal strictures. However, in infants with NEC that was managed surgically (stage III), anaerobic antimicrobial therapy was associated with lower mortality. These results suggest that anaerobic coverage is beneficial in cases of severe and perforated NEC (stage III).

In summary, there is insufficient evidence to support one antibiotic regimen over another for the treatment of NEC. Empirical therapy should be effective against most pathogenic bacteria usually present in the intestinal flora (e.g., *Enterobacteriaceae* and gram-positive cocci). Clinicians should also consider local resistance rates, individual patients' microbiologic results, and clinical evolution to guide their antibiotic choice. Given that broad-spectrum antibiotics (including 3rd generation cephalosporins and carbapenems) increase the risk of adverse outcomes (IC, increased risk of colonization with antimicrobial-resistant organisms, and microbiota alterations), narrow-spectrum regimens and short therapy durations are encouraged.

There is no evidence supporting the optimal duration of antibiotic therapy for NEC (189). In stage I and stage IIA NEC (modified Bell's stage) (184), experts are generally in favor of discontinuing antibiotics after 3 days and after 5–7 days, respectively, if symptoms are resolved. In higher-stage NEC (IIB and III) or when the infant presents with bacteremia or sepsis, the duration of the antibiotics is generally extended to 10–14 days. If an intra-abdominal abscess has been identified, antibiotics should be continued until clinical and radiological responses are established. The recurrence rate of NEC after the first course of antimicrobial or surgical treatment does not appear to be reduced by a longer duration of treatment (200).

Finally, NEC is a risk factor for invasive candidiasis (IC), and fluconazole prophylaxis may be beneficial for high-risk infants. A study in very low BW infants, <6 weeks postnatal age, receiving broad-spectrum antibiotics for >2 days with 1 additional risk factor including NEC, showed that fluconazole prophylaxis reduced the incidence of IC, but failed to demonstrate a survival benefit (201).

### Practice Summary

- In infants with Bell stage I or suspected NEC, consider parenteral antibiotics such as ampicillin and gentamicin for 48 to 72 h pending cultures and evolution.
- In infants with Bell stage II or III NEC, therapy may include the following parenteral antibiotic regimens:
  - Ampicillin and gentamicin +/- metronidazole or clindamycin;
  - Piperacillin-tazobactam as a single agent, especially for more severe presentation.
  - Anaerobic coverage may be added for more severe presentation (stage IIB and III NEC)
- In infants with Bell stage II NEC, antibiotics may be discontinued after 5–7 days if symptoms are resolved.
- In infants with Bell stage III NEC or when the infant presents with bacteremia or sepsis, the duration of the antibiotics should be 10–14 days.

## Urinary Tract Infection

Urinary tract infections (UTIs) are significant bacterial infections in neonates. The incidence and prevalence of neonatal UTIs range from 3–25% in preterm and 0.1–20% in term infants (202–207). Clinical presentation of neonatal UTIs is non-specific and may include the following: poor feeding, failure to thrive, vomiting, diarrhea, prolonged jaundice, lethargy, irritability, hypothermia, hypoglycemia, abdominal distention, and bradycardic events (204–206). Over 50% of premature infants present with respiratory symptoms including apnea, hypoxia, or tachypnea (206). Risk factors include male sex, being uncircumcised, prematurity, VLBW (<1,500 g), renal/urinary tract malformations, and prolonged hospitalization (202–209). Urine collection should be part of LOS evaluations.

### Microbiology

The most common pathogen found in neonatal UTIs is *E. coli*, followed by *Klebsiella pneumoniae* and *Enterobacter spp* (204–206, 210, 211). Less common pathogens include: *Pseudomonas aeruginosa, Enterococcus spp*, Group B *Streptococcus, S. aureus*, viridans group *Streptococci, Citrobacter freundii, Serratia marcescens, Klebsiella oxytoca*, and *Proteus vulgaris* (205, 206, 210). Neonatal UTIs caused by fungal pathogens, specifically *Candida* species, could be associated with higher mortality (204, 208, 212, 213). Patients with vesicoureteral reflux may present with less common pathogens and are predisposed to pyelonephritis (204, 205, 211).

### Sample Collection

Suprapubic aspiration is the recommended method, but it is an invasive procedure that requires operators with skills in point-of-care ultrasound (POCUS) (206, 214, 215). Urinary catheterization has also been shown to be feasible in preterm infants, even with 24 weeks gestation, although it can also be technically difficult (214). Proper disinfection of the penis or perineum is essential to minimize the possibility of sample contamination. Sterile bag collection is not recommended because of its high contamination rate (204–206).

### Diagnostic Definitions

The thresholds for laboratory values diagnostic for UTIs vary across literature. The current gold standard for UTI diagnosis is a urine sample obtained by suprapubic aspiration or urinary catheterization that is positive for a single pathogenic organism (204–206). The most accepted threshold of pathogen growth is a concentration of ≥5x10^7^CFU/L (211). However, some studies have included a threshold of ≥10^7^CFU/L, especially in the presence of pyuria, to define a positive culture (204–206).

Elevated CRP and erythrocyte sedimentation rates were reported to have low sensitivity and specificity among neonates with UTI (204–206).

### Antimicrobial Treatment—Choice and Duration

The initial treatment for neonatal UTI involves empirical parenteral broad-spectrum antibiotics (204–206). Most commonly, a combination of ampicillin and gentamicin or ampicillin and cefotaxime is administered to cover *Enterococcus spp* and gram-negative bacilli (204–206). Once a pathogenic organism is identified, treatment is tailored based on antimicrobial sensitivities (204–206). There is currently a lack of evidence on the optimal treatment duration. Traditionally, neonates were treated intravenously for 5–7 days (204–206). However, there is increasing evidence that term infants can complete therapy with oral antibiotics (216). The use of antibiotic prophylaxis for neonates with recurrent UTIs is not supported by evidence (205, 206).

### Sequelae and Associations

The risk of detecting bacteremia in an infant with a UTI is moderate, ranging from 3–17%, and is inversely related to age (204, 205, 211, 217). Renal ultrasonography is recommended for all neonates after the first episode of an UTI because of the high incidence of associated congenital anomalies in this population (204–206, 211, 218, 219). Early diagnosis of urological abnormalities is critical for managing recurrent infections and preventing renal scarring (205, 212).

### Practice Summary

- The clinical presentation of neonatal UTIs is often non-specific.
- Obtaining a urine sample for culture should be considered in LOS evaluations for neonates. Suprapubic tap should be attempted if expertise exists and especially if POCUS is available. Meticulous site disinfection is essential when obtaining a catheter sample to minimize contamination.
- A combination of ampicillin and gentamicin or ampicillin and cefotaxime can be administered to provide empirical coverage for UTI, although local resistance patterns should be considered.
- There is currently a lack of evidence regarding the optimal treatment duration for neonatal UTI. Most studies recommend a treatment duration of 5–7 days.

## Surgical Prophylaxis

Surgical site infections (SSIs) involve the skin, subcutaneous, or deep tissue of an area of surgical intervention within 30 days of surgery (220). These are one of the most common healthcare-associated infections (221) and are an important cause of morbidity in the NICU (222). The reported incidence of SSI in infants in the NICU ranges from 4.7 (222) to 11.7% (223). Prophylactic antibiotics (given within 60 minutes of skin incision to mitigate the risk of SSI) may be unnecessary, overly broad spectrum, or given inappropriately for prolonged periods (224). Inappropriate antibiotic use drives antimicrobial resistance. In the NICU, it has been associated with altered intestinal microbiota, increased risk of IC, and LOS, and in VLBW infants, NEC and death (16–18, 225, 226). Although guidelines exist to aid clinicians with the timing, class, and duration of antimicrobial prophylaxis, there is a paucity of evidence for the NICU (227, 228). Institutional guidelines for SSI prophylaxis can help reduce the rates of infection while limiting adverse effects from inappropriate antimicrobials administration (224) (Table 2).

**Table 2:** Prophylaxis recommendations for surgical site infections in neonates [adapted from Laituri et al. (228)].

**Wound classification**	**Examples of procedures**	**First choice of antibiotics**	**Penicillin** **Allergy**	**Initial dose timing**	**Post-op** **duration**	**Citation**
**Clean (class 1)**	PDA ligation, CVL insertion, inguinal hernia, circumcision. Exceptions: CDH repair, gastroschisis reduction, omphalocele.	None	NA	NA	NA	(228, 230–232)
**Clean-contaminated (class 2)**	Duodenal atresia repair, gastrostomy (G)-tube insertion, choledochal cyst excision. Exceptions: laparoscopy pyloromyotomy, colorectal surgery	cefazolin IV		Within 1 h of incision	None	(228)
**Contaminated (class IV)**	Perforated NEC	See NEC guidelines				
**Specific cases**	Gastroschisis	Ampicillin or cloxacillin + gentamicin	Cloxacillin + gentamicin	At birth	Discontinue after wound closure	(228, 235)
**Specific cases**	Colorectal surgery	Cefazolin + metronidazole		within 1 h of incision	Discontinue within 24 h of surgery	(241, 242)

### Clean (Class 1)

Clean procedures—an incision in which no inflammation is encountered in a surgical procedure, without a break in sterile technique, and during which the respiratory, alimentary, and genitourinary tracts are not entered—include the insertion of a central venous line (CVL), patent ductus arteriosus (PDA) ligation, immediate repair of omphalocele, gastroschisis reduction, repair of congenital diaphragmatic hernia (CDH), inguinal hernia repair, and circumcision (228, 229). Data on the need for prophylactic antibiotics for peripherally-inserted central catheter placement is mixed (230–232). While some RCTs demonstrated reduced catheter-related bloodstream infection (230) and CoNS catheter-related sepsis, (231) others failed to show benefit (232). The rates of SSI with CVL can be as low as 0 per 100 procedures; therefore, the risks of antimicrobial exposure are unlikely to outweigh the benefits (222, 230).

Although omphalocele is considered a clean procedure, short-term preoperative antibiotics are recommended for primary closure of omphalocele, or antibiotics are continued until closure in staged repairs (233, 234).

Despite its classification as a clean procedure, wound infections in gastroschisis repair are common, leading to the argument that this procedure should be re-classified based on a higher level of contamination (222). Wound infections were more common in neonates undergoing delayed closure (21%) than in those undergoing immediate closure (8.2%) (235). Empiric coverage with a beta-lactamase-resistant penicillin derivative (i.e., cloxacillin) combined with an aminoglycoside initiated at birth and discontinued after wound closure is suggested (235).

Retrospective data demonstrated that SSI is an uncommon source of infection in children with congenital diaphragmatic hernia (CDH), occurring in less than 1% of 1,085 neonates, but prophylactic antibiotics are typically prescribed (236, 237).

There are no specific neonatal guidelines on SSI prophylaxis for PDA ligation, inguinal hernia, or circumcision, but apart from the above exceptions, prophylactic antibiotics are not recommended for clean procedures based on the expert opinion (228).

### Clean-Contaminated (Class 2)

This class describes an operative wound in which the respiratory, alimentary, genital, or urinary tract is entered under controlled conditions and without unusual contamination. Clean-contaminated surgical procedures include esophageal atresia (EA), tracheoesophageal fistula (TEF) repair, duodenal atresia repair, gastrostomy G-tube insertion, choledochal cyst excision, and colorectal surgery.

A small retrospective review of 732 neonatal surgeries found no statistical difference in SSI among infants receiving greater than 24 h of postoperative antibiotics and those receiving less than 24 h for clean contaminated or contaminated gastrointestinal operations. Guidelines recommend a single dose of preoperative intravenous (IV) cefazolin prior to most clean-contaminated procedures (228).

A consensus on the role of antimicrobial prophylaxis for EA and TEF repair is lacking (228). A study on 48 patients with EA, isolated either normal oropharyngeal organisms or no organisms from the upper esophageal pouch of infants who underwent repair within the first 24 h despite half of them being on prophylactic antibiotics (238). All infants who underwent delayed closure demonstrated growth of micro-organisms, irrespective of whether they were on antibiotics, and only those on prophylactic antibiotics demonstrated growth of *Pseudomonas* and *Serratia* (238). Antibiotic prophylaxis for EA/TEF repair is often recommended with amoxicillin-clavulanate, or 24–48 h of cefazolin and metronidazole, piperacillin/tazobactam, or cefoxitin (228) in the absence of evidence of aspiration (238–240).

Despite a lack of pediatric data, the Surgical Care Improvement Project (SCIP) recommends SSI prophylaxis in children undergoing colorectal surgery (241, 242). This recommendation is based on a Cochrane review and meta-analysis of adult studies demonstrating significantly improved rates of SSI when prophylactic antibiotics were used compared to when no treatment was provided [risk ratio (RR): 0.34] (241, 242). There was a further 50% reduction in SSI when anaerobic coverage was added to agents targeting aerobic bacteria (RR: 0.55), and a 60% reduction when aerobic coverage was added to agents targeting anaerobes (RR: 0.41) (241, 242). SCIP guidelines recommend parenteral cefazolin + metronidazole within 1 h of incision and their discontinuation within 24 h of surgery completion (241).

### Contaminated (Class 3)

There are no common class 3 procedures in the NICU population.

### Contaminated Perforated Necrotizing Enterocolitis (Class 4)

Antimicrobial recommendations for NEC are covered in Section 5 of this narrative review.

### Practice Summary

- Prophylactic antibiotics have not been shown to reduce SSI in neonates undergoing clean procedures (class I).
- Prior to most clean-contaminated procedures (class II), a single dose of preoperative cefazolin IV is suggested.
- For colorectal surgeries, 24 h of antimicrobial coverage with cefazolin and metronidazole is recommended as SSI prophylaxis.
- For the management of gastroschisis, empiric coverage with a beta-lactamase-resistant penicillin derivative combined with an aminoglycoside is recommended for SSI prophylaxis.
- More high-quality evidence is needed to guide the use of antimicrobials for SSIs in the NICU.

## Conclusion

Antimicrobials are the most frequently used medications for neonates, yet the untoward side effects of unnecessary antimicrobial exposure need to be acknowledged. The NICU houses immunocompromised newborns who are highly susceptible to overwhelming infections, but at the same time, there is a lack of high-quality evidence on the definition and optimal antimicrobial management of infection-related conditions in these vulnerable populations. This narrative review summarizes the best practice points based on the existing yet limited literature.

Further research is required to promote the judicious use of antibiotics based on the best practices and to elucidate the most impactful stewardship interventions for very preterm infants. This can only be achieved by national collaborations to develop consensus around definition, management approaches, and quality improvement efforts to enhance the health outcomes of neonates in both the short- and long-term.

## Future Direction of Research

Neonatal infection can result in significant short- and long-term adverse outcomes. Current practices are often based on expert consensus, because of lack of well-powered trials and the high risk of systematic errors. The definitions of some conditions and treatment recommendations are extrapolated from the pediatric or even adult studies, without proper validation in the neonatal populations.

Large randomized controlled trials assessing different antibiotic regimens in early- and late-onset neonatal sepsis with low risk of bias are warranted. Further exploration on the use of sepsis risk calculator in the initiation and cessation of antibiotic use, particularly among those born at <35 weeks GA is needed. Development of specific standardized diagnostic criteria for neonatal UTI and VAP is urgently needed, before one can evaluate their treatment outcomes properly. Study on the correlation of antibiotic treatment strategies with bowel recovery and other clinically relevant outcomes are lacking. The optimal management of “mild” NEC cases picked up by ultrasound but not the traditional radiographic and clinical criteria needs further studies. Further evaluation of the best surgical prophylaxis for infants undergoing surgeries with relatively new techniques like flap closure for gastroschisis and bronchoscopic repair of thoracic conditions are necessary.

## Author Contributions

JT, JA, MD, AR, K-sL, JR, and PS contributed to conception of the review. JT, JA, SG-B, KA, SK, JL, GL, FM, and IV-T wrote the first draft of the manuscript. All authors contributed to manuscript revision, read, and approved the final version.

## Funding

This work was supported by the Canadian Institutes of Health Research (201903PJT-420294-CA2-CAAA-245530), which has no role in the development of this review and the writing of manuscript.

## Conflict of Interest

The authors declare that the research was conducted in the absence of any commercial or financial relationships that could be construed as a potential conflict of interest.

## Publisher's Note

All claims expressed in this article are solely those of the authors and do not necessarily represent those of their affiliated organizations, or those of the publisher, the editors and the reviewers. Any product that may be evaluated in this article, or claim that may be made by its manufacturer, is not guaranteed or endorsed by the publisher.
