# Supplementary material for: Practice Summary of Antimicrobial Therapy for Commonly Encountered Conditions in the Neonatal Intensive Care Unit: A Canadian Perspective
Source: Front Pediatr. 2022 Jul 8;10:894005. doi: 10.3389/fped.2022.894005 (PMC9304938; doi:10.3389/fped.2022.894005)
Supplement: Supplementary file 1 [file Data_Sheet_1.docx]

List of Abbreviations (alphabetical)

| ASP | antimicrobial stewardship program |
| --- | --- |
| BAL | bronchioalveolar lavage |
| BW | birth weight |
| CBC | complete blood count |
| CD64 | cluster of differentiation 64 |
| CDC | Center for Disease Control and Prevention |
| CDH | congenital diaphragmatic hernia |
| CFU | colony-forming units |
| cIAI | complicated intra-abdominal infections |
| CLABSIs | central line–associated bloodstream infections |
| CNN | Canadian Neonatal Network |
| CoNS | coagulase-negative *Staphylococcus* |
| CPS | Canadian Pediatric Society |
| CRP | C-reactive protein |
| CSF | cerebrospinal fluid |
| CVL | central venous line |
| EA | esophageal atresia |
| ELBW | extremely low birth weight |
| EOS | early-onset sepsis |
| EPIQ | Evidence-based Practice for Improving Quality |
| ESBL | extended-spectrum β-lactamases |
| GA | gestational age |
| GBS | group B *streptococcus* |
| HAI | healthcare-associated infection |
| IAP | intra-partum antimicrobial prophylaxis |
| IC | invasive candidiasis |
| IDSA | Infectious Diseases Society of America |
| IFN-γ | gamma interferon |
| IL-6 | interleukin 6 |
| IL-8 | interleukin 8 |
| IV | intravenous |
| IVAC | infection-related ventilator-associated complication |
| LOS | late-onset sepsis |
| LP | lumbar puncture |
| MDR | multidrug-resistant |
| MDRO | multi-drug resistant organisms |
| MRSA | methicillin-resistant *Staphylococcus* *aureus* |
| NEC | necrotizing enterocolitis |
| NHSN | National Health and Safety Network |
| NICU | neonatal intensive care unit |
| PCR | polymerase chain reaction |
| PDA | patent ductus arteriosus |
| POCUS | point-of-care ultrasound |
| PROM | premature rupture of membranes |
| RCT | randomized controlled trials |
| ROM | rupture of membranes |
| RR | risk ratio |
| SCIP | Surgical Care Improvement Project |
| sICAM | soluble intercellular adhesion molecule |
| SOGC | Society of Obstetricians and Gynaecologists of Canada |
| SRC | sepsis risk calculator |
| SSI | surgical site infection |
| TA | tracheal aspirates |
| TEF | tracheoesophageal fistula |
| TNF-α | tumor necrosis factor alpha |
| UTI | urinary tract infection |
| VAC | ventilator-associated condition |
| VAP | ventilator-associated pneumonia |
| VLBW | very-low-birth-weight |
